# Identifying optimal reference genes for gene expression studies in Eurasian spruce bark beetle, *Ips typographus* (Coleoptera: Curculionidae: Scolytinae)

**DOI:** 10.1038/s41598-022-08434-3

**Published:** 2022-03-18

**Authors:** Gothandapani Sellamuthu, Jan Bílý, Mallikarjuna Reddy Joga, Jiří Synek, Amit Roy

**Affiliations:** 1 Excellent Team for Mitigation (ETM), Faculty of Forestry and Wood Sciences, Czech University of Life Sciences Prague, Prague, Czech Republic; 2 EVA 4.0 Unit, Faculty of Forestry and Wood Sciences, Czech University of Life Sciences Prague, Prague, Czech Republic

**Keywords:** Biological techniques, Gene expression analysis, Reverse transcription polymerase chain reaction

## Abstract

Eurasian spruce bark beetle (*Ips typographus* [L.]) causes substantial damage to spruce forests worldwide. Undoubtedly, more aggressive measures are necessary to restrict the enduring loss. Finishing genome sequencing is a landmark achievement for deploying molecular techniques (i.e., RNA interference) to manage this pest. Gene expression studies assist in understanding insect physiology and deployment of molecular approaches for pest management. RT-qPCR is a valuable technique for such studies. However, accuracy and reliability depend on suitable reference genes. With the genome sequence available and the growing requirement of molecular tools for aggressive forest pest management*,* it is crucial to find suitable reference genes in *Ips typographus* under different experimental conditions. Hence, we evaluated the stability of twelve candidate reference genes under diverse experimental conditions such as biotic (developmental, sex and tissues) and abiotic factors (i.e., temperature and juvenile hormone treatment) to identify the reference genes. Our results revealed that *ribosomal protein 3a* (*RPS3-a*) was the best reference gene across all the experimental conditions, with minor exceptions. However, the stability of the reference gene can differ based on experiments. Nevertheless, present study provides a comprehensive list of reference genes under different experimental conditions for *Ips typographus* and contributes to “future genomic and functional genomic research”.

## Introduction

The Eurasian spruce bark beetle, *Ips typographus* (Coleoptera: Curculionidae: Scolytinae), is the most devastating pest in Eurasian coniferous forests of Norway spruce (*Picea abies* (L.) Karst) and has caused significant ecological and economic damage in recent years under the influence of climate change. It primarily affected European countries^1–4^ and was recently found in Kent and East Sussex (England) (Forestry Commission England, 2021, https://www.gov.uk/government/news/forestry-commission-acts-on-bark-beetle-tree-pest). Some recent publications even indicated the risk of *Ips typographus* and its associated pathogen invasion to Canada and America based on climatic and host suitability^5,6^. In Europe, tree mortality exceeded 2.9 million m^3^ per year from 1950 to 2000 and has increased further in the last decades^7–9^. In the Czech Republic, the loss of vegetation was estimated to be 14.5 million m^3^ during recent years^10^. *Ips typographus* thrives on dying trees when their populations are at the endemic stage, and mass attack on healthy trees happens only at epidemic levels^1,11,12^. However, climate change often rises the occurrence and severity of outbreaks^9,13–15^. The management of *I. typographus* has been primarily relied on mass-trapped using pheromone, sanitary cutting, trap trees, salvage logging, and insecticides^16^. Protecting the forest health and environment from toxic chemical insecticides has led to searching for superior alternatives for forest pest insect management strategies such as RNAi, as reviewed ^17^. Therefore, molecular studies are essential for facilitating new pest management strategies using available genomic resources.

Gene expression analyses have become immensely important for revealing gene function and molecular regulation under the different environmental responses during the bark beetle life cycle or beetle-host interaction. Reverse transcription-quantitative polymerase chain reaction (RT-qPCR) has become the most extensively accepted methodology for detecting and quantifying target gene expression with higher accuracy, sensitivity, and reproducibility compared with other traditional methods molecular techniques^18–23^. It is especially useful to detect low-abundance mRNAs in limited samples^24,25^. Nevertheless, RT-qPCR data are influenced by many factors such as initial RNA sample quantity and quality, the efficiency of cDNA synthesis, mRNA recovery, primer and PCR efficiency^26–31^. Furthermore, the reliability of RT-qPCR data is highly dependent on the appropriate reference genes as internal controls from the same samples across various biotic and abiotic stresses and treatments^25^. A literature search indicates that reference genes that are constitutively expressed under different environmental factors and maintain the essential cellular functions have been used extensively as internal controls for expression normalization of target genes^32,33^. It is pretty clear now that a single reference gene is not appropriate for the more comprehensive experimental conditions, and it can generate an error in the gene expression estimations causing nonoptimal interpretation of the data^25,34,35^. For mitigation, it is often recommended to use multiple reference genes to minimize variations by RT-qPCR normalization^23,36,37^. Several research studies revealed that most reference gene expression depends on samples/experimental conditions, suggesting no universal reference gene is available for all experimental conditions^38^. Hence, for accurate gene expression normalization, it is essential to evaluate the stability of reference genes for different environmental conditions, life stages, sex-specific and tissue-specific stages for each insect^25,31,32,39–43^.

In the present study, we analyzed the expression level of 12 commonly occurring reference genes in different coleopteran insects based on published articles^38,43,44^. These genes mostly perform conserved cellular functions in the coleopteran insects, hence expected to be constitutively and stably expressed in all tissues and cells under different experimental conditions^38^. Our objective is to find suitable reference genes for future gene expressions studies in *Ips typographus*. Candidate reference genes were evaluated across different experimental conditions are *elongation factor 1α* (*EF-1α*)*, ribosomal protein L13a* (*RPL13a3*)*, arginine kinase isoform X1*(*ArgK*)*, ribosomal protein L7* (*RPL7*)*, glyceraldehyde-3-phosphate dehydrogenase* (*GAPDH*)*, β-actin* (*Actin*)*, ribosomal protein 3a* (*RPS3-a*)*, Tubulin beta-1 chain* (*β-Tubulin*)*, ubiquitin C variant* (*UbiQ*)*, V-type proton ATPase catalytic subunit A* (*V-ATPase-A*), *ribosomal protein S7* (*RPS7*)*, ribosomal protein L6* (*RPL6*). Reference genes were tested across developmental stages (first, second and third instar larvae, pupa, adult male and female) and tissues (head, midgut, and fat body). Additionally, adult beetles were exposed to juvenile hormone treatment (JHIII) and varied temperatures to identify reference genes for those conditions. Wild and lab-reared beetles were also evaluated for the same intent. Nevertheless, the present study tried to identify reference genes suitable for various tested experimental conditions for *I. typographus*.

## Material and methods

### Beetles

*Ips typographus* were obtained from Kostelec nad Cernými lesy (50° 00′ 07.2″ N 14° 50′ 56.3″ E, under School Forest Enterprise) in summer 2021. School Forest Enterprise in Kostelec nad Černými lesy (Czech University of Life Sciences Prague) is located in the Central Bohemia region, 40 km southwest from Prague. It is a relatively warmer and dryer area, mean annual temperature of 7–7.5 °C, the mean annual sum of precipitation 600 mm, length of vegetation season 150 days. Range of elevation 350–520 m a.s.l. Natural vegetation was composed mainly of beech and oak mixed forest with fir. However, the current species composition is dominated by spruce (50%) and pine (18%)^45^. Due to the extreme drought in 2018, the whole area was under tremendous pressure from the bark beetle outbreak, mainly *I. typographus*^46^. Collected beetles from the forest were maintained in an insect rearing chamber with fresh Norway spruce logs (*Picea abies*) at 27 ± 1 °C under 70 ± 5% humidity and a 16:8-h light/dark (L:D) photoperiod at Faculty of Forestry and Wood Sciences, Czech University of Life Sciences, Prague. The wild beetle population was supplied with fresh spruce logs for the next generation and maintained for 35–40 days.

### Experimental conditions

#### Biotic factors (life stages and tissue types)

**Figure 1:**
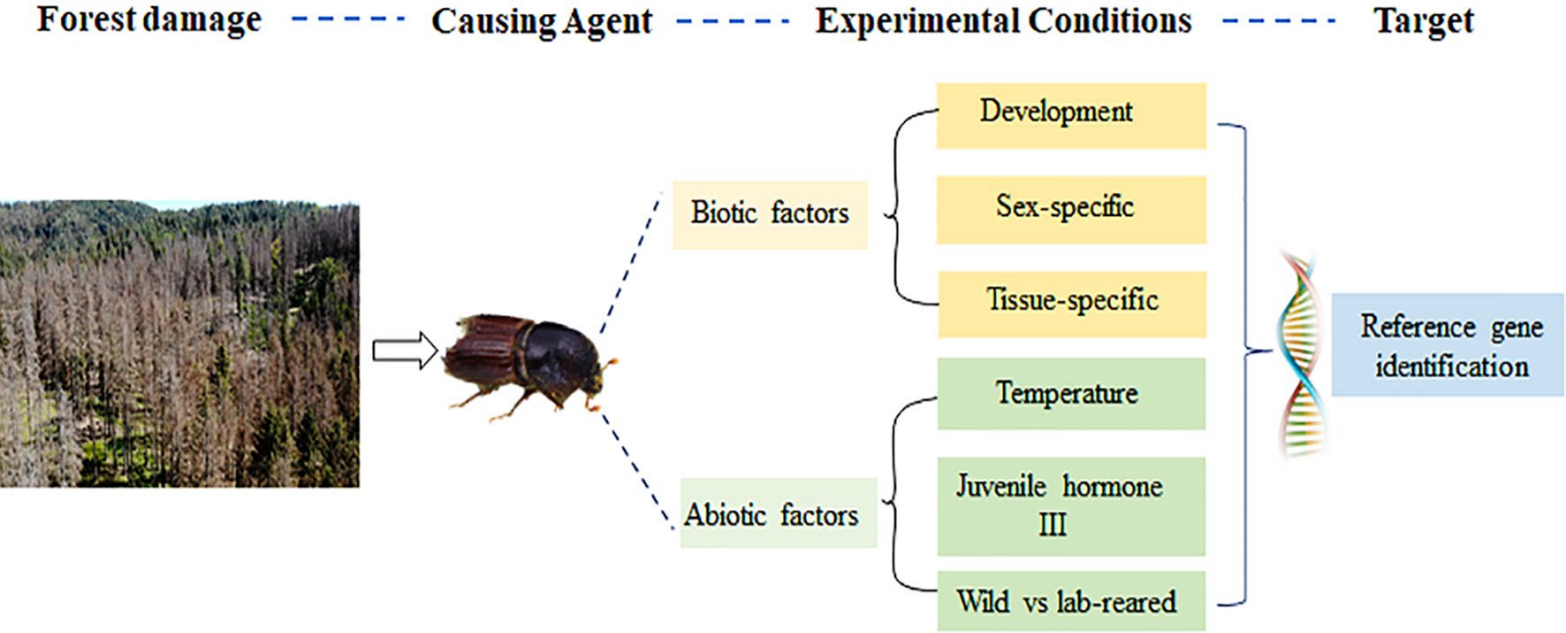
Schematic representation of experimental plan for finding reference genes in *Ips typographus*.

Developmental stages such as three larval stages, pupae, callow male and female, and fed adult male and female were collected from *I. typographus*. Tissues, including head, fat body, and gut, were dissected from the callow and fed adult males and females (Fig. 1). The samples details are summarized in Table 1. Each replicate was derived by pooling tissues from ten beetles for tissue-specific comparisons. Four biological replicates were used for each bark beetle developmental stages and tissues.

**Table 1:** Detailed sample list of *I. typographus* life stages and tissue types used for reference gene experiments.

Sample category	Sample name	Abbreviation
Developmental stages	Larval 1	L1
	Larval 2	L2
	Larval 3	L3
	Pupae	P
	Callow male	CM
	Callow female	CF
	Fed adult male	AMF
	Fed adult female	AFF
Tissues	Callow male head	CMH
	Callow male gut	CMG
	Callow male fat body	CMFB
	Callow female head	CFH
	Callow female gut	CFG
	Callow female fat body	CFFB
	Fed adult male head	AMFH
	Fed adult male gut	AMFG
	Fed adult male fat body	AMFFB
	Fed adult female head	AFFH
	Fed adult female gut	AFFG
	Fed adult female fat body	AFFFB

#### Abiotic factors (temperature, JHIII treatment and wild beetles vs lab-reared beetles)

To examine the effect of temperature treatment, the freshly emerged adults were exposed to 4, 27, and 37 °C for 72 h. For the juvenile hormone treatment (JHIII), fed adult male and female beetles were treated topically on the ventral surface of the abdomen with 10 µg JHIII (Sigma-Aldrich, St. Louis, MO, USA) and acetone as control^47,48^. Beetles were maintained in similar conditions as mentioned above for 72 h. Wild beetles (adults) were collected from the infested Norway spruce stands from Kostelec nad Cernými lesy, Czech Republic, to examine the climatic effect. Similarly, laboratory-reared adults emerged from eggs produced by the wild- beetles. The lab-reared beetles were collected after three generations. Four independent biological replications for each experimental condition were used. The experimental design is summarized in Fig. 1.

### Selection of candidate reference genes for evaluation

The reference genes selected for this study were reference genes in other Coleoptera species and showed stable expression in our in-house *I. typographus* genome data^44^ (Data not shown). The primers for those selected genes were designed via IDT (Integrated DNA Technologies), and the primer efficiencies (E) and correlation coefficients (R2) were also calculated (Table 2).

**Table 2:** Primer sequence, amplicon length and RT-qPCR analysis of candidate reference genes and a target gene.

Gene symbol	Gene name	BioProject accession numbers PRJNA671615*	Primer sequences (5′–3′)	Amplicon length (bp)	PCR efciency %	Regression coefcient
*EF1-a*	Elongation factor 1-alpha	Ityp13440	**FP**: GCCATTCTTCCACCATCTCGTC	210	98.99	0.989
			**RP**: CAGGAGCAAACACCACTACCATAC			
*RPL13a*	60S ribosomal protein L13a	Ityp07303	**FP**: CTGGAGCACTTAGGGTTGTC	215	97.2	0.972
			**RP**: TGAATGGCTTCACCTGCTTG			
*ArgK*	Arginine kinase	Ityp12981	**FP**: GTCTTCGCTGACTTGTTCGACC	104	119.24	0.985
			**RP**: GCCAAATACGCTCACATCACCC			
*RPL7*	60S ribosomal protein L7	Ityp01351	**FP**: GCTGAACACCCCAACTGGAG	100	97.6	0.976
			**RP**: CTGTTGCCAAAGTCACCACC			
*GAPDH*	Glyceraldehyde-3-phosphate dehydrogenase	Ityp02436	**FP**: GGCAAATTGTGGAGAGAGGGAC	200	96.9	0.969
			**RP**: GGCGGCTTCCTTGATCTTGG			
*Actin*	β-actin	Ityp12788	**FP**: ACGAAAGATTCCGTTGCCCC	170	114.79	0.954
			**RP**: GCGTACAAGTCCTTACGAATGTCC			
*RPS3-a*	40S ribosomal protein S3-A	Ityp04549	**FP**: GCCCCTTCAATGTTTGCCAC	119	96.2	0.972
			**RP**: AAGTCCGCCAAACTAACCTCA			
*β-Tubulin*	Tubulin beta-1 chain	Ityp08900	**FP**: TGATGACGAGTACGAAGCGG	138	114.67	0.984
			**RP**: CAAAGCAAGGCACTCTTGGTC			
*UbiQ*	Ubiquitin C variant	Ityp20676	**FP**: CGCACACAAATCATGGGTGG	120	94.1	0.944
			**RP**: GTCAAAGGCACGTCTGATTTC			
*V-ATPase-A*	V-type proton ATPase catalytic subunit A	Ityp14486	**FP**: GTCGCCTCCTACAGGGAATG	120	92.2	0.922
			**RP**: CCAGAGACCGAAGGCAAACA			
*RPS7*	40S ribosomal protein S7	Ityp08836	**FP**:CTGGTTAGGGAGTTGGAGA	140	118.48	0.969
			**RP**:GCATCATATACAGCCGTCAG			
*RPL6*	60S ribosomal protein L6	Ityp04817	**FP**:CCACCAGCGAACATTGAG	148	115.94	0.971
			**RP**: TTCCGTAGGGACCTGTAAC			
**Target Gene**						
*CYP03903*	Cytochrome P450	Ityp03903	**FP**:GTATTCGCCTGCTCATTCC	114	102.44	0.951
			**RP**:CCGGTCTACTGGATCTGTT			

*Powell et al. 2021.

### Total RNA extraction and RT-qPCR analysis

Total RNA was extracted from each tissue using TRIzol® (Invitrogen, Carlsbad, CA) following the manufacturer's protocol. Isolated RNA was further treated with DNases using a TURBO DNAase Kit (Ambion, USA). cDNA was synthesized from 1 μg RNA using the High-Capacity cDNA Reverse Transcription kits (Applied Biosystems-Life Technologies) following the manufacturer's recommendations and stored at − 20 °C. cDNA samples were diluted ten times before RT-qPCR reaction. Four independent biological replicates from each treatment were included in each RT-qPCR experiment. RT-qPCR reaction mix contained 5.0 µL of SYBR® Green PCR Master Mix (Applied Biosystems), 1.0 µL of cDNA, 1.0 µL optimized concentrations of primers (Table 2), and RNase-free water (Invitrogen) to a total volume of 10.0 µL. Amplification conditions were as follows: initial denaturation at 95 °C for 10 min, followed by 40 cycles of 95 °C for 15 s, 60 °C for 1 min. The reactions were performed in an Applied Biosystems™ StepOne™ Real-Time PCR System (Applied Biosystems). To confirm the primer specificity, melting curve analysis was used to confirm gene-specific amplification by a steady increase in temperature from 60 to 95 °C. All RT-qPCR assays were carried out in four biological replicates, including three technical replicates.

### Data analyses

Four different algorithms, namely geNorm^25^, NormFinder^49^, BestKeeper^39^ and ΔCT method^50^, were used for measuring reference gene expression stability. geNorm computes the expression stability value (M) and pairwise variation comparison. NormFinder ranks the set of reference genes based on their expression stability within a given set of samples. BestKeeper is also a freely available algorithm that considers the Cq (quantification cycle) value of all reference genes to evaluate standard deviation and correlation coefficient. ΔCT method directly evaluates the relative expression of 'gene pairs' within each sample. The mean Cq values of each reference gene from each experiment are offered as input data and subsequently processed using the web-based tool RefFinder (https://www.heartcure.com.au/reffinder/), which delivers a comprehensive stability index that ultimately ranks each reference gene. Pairwise variation (V), estimated by geNorm, was used to determine the optimal number of reference genes for precise RT-qPCR normalization. The Vn/Vn + 1 value exhibited the pairwise variation between two sequential normalization factors^25^.

### Validation of selected reference genes

In insects, cytochrome P450 monooxygenases (P450s) are key enzymes that detoxify a broad spectrum of xenobiotics such as plant allelochemicals and synthetic insecticides. To validate the selected reference gene, we analysed relative expression levels of cytochrome P450 (*CYP03903*; Table 2) in life stages and various tissue types of the beetle according to the 2 − ΔΔCT method for gene normalization using most and least stable reference genes^51^. Target genes mRNA expression level was analysed using one-way ANOVA using GraphPad Prism software. *P*-value < 0.05 was deployed as the cut-off to indicate significant differences between tested samples.

## Results

### Candidate reference gene selection and PCR efficiency

**Figure 2:**
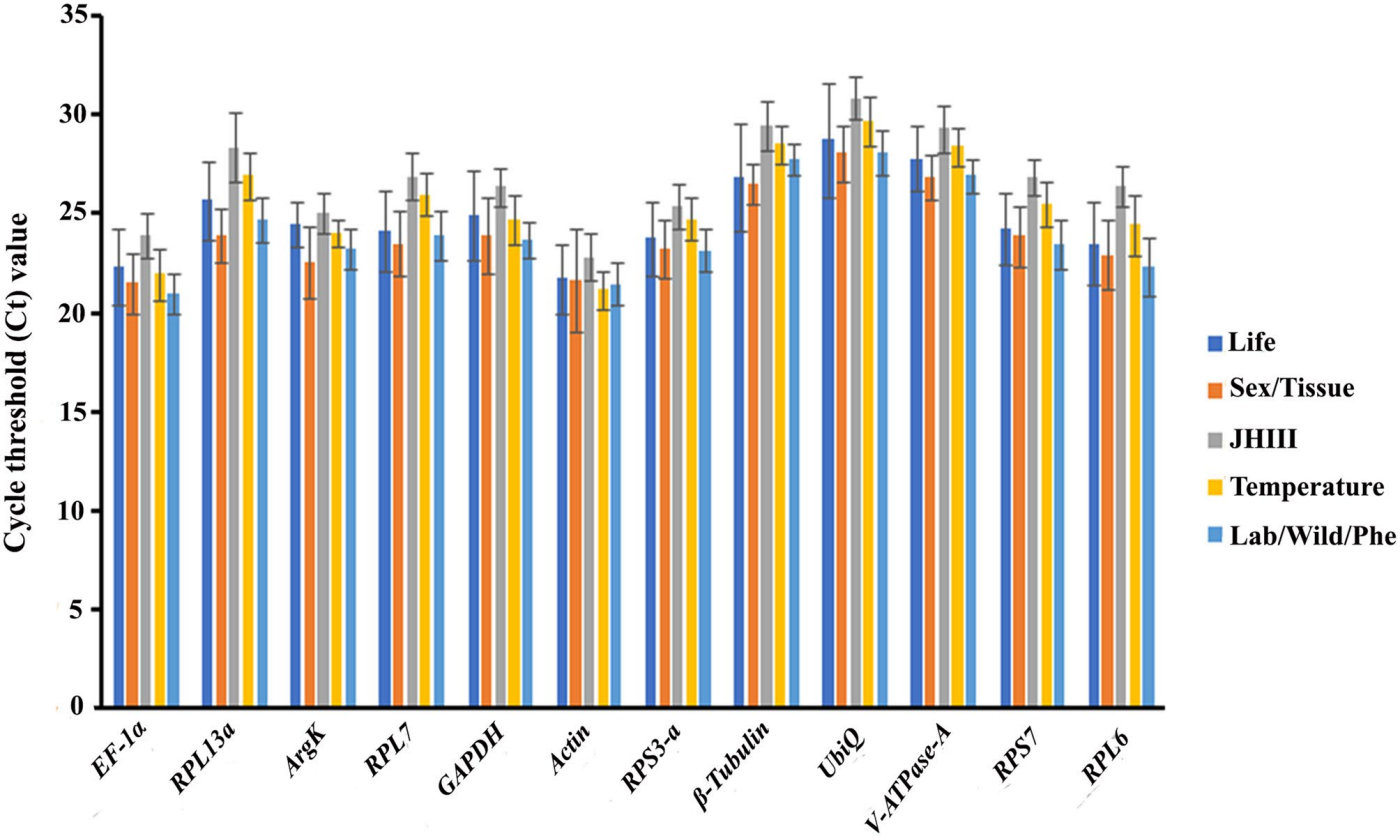
Expression range of Ct (Cycle threshold) values of candidate reference genes under different experimental conditions in *I. typographus*. The different conditions included in biotic conditions: developmental stages (i.e., larvae, pupae, and adult stages), sex-specific (male and female), tissue types (head, midgut, fat body) and abiotic conditions: different treatments (such as temperature; Juvenile hormone III; wild-collected vs long-term laboratory-reared beetles).

Total twelve candidate reference genes, *EF-1α, RPL13a, ArgK, RPL7, GAPDH,Actin, RPS3-a, β-Tubulin, UbiQ, V-ATPase-A, RPS7,* and *RPL6* were selected for identifying suitable reference genes from *I. typographus*. Each reference gene was produced a single amplicon, as deducted by agarose gel electrophoresis (Figure S1) and melting curve analyses (Figure S2). The amplification efficiency of each primer pair fluctuated from 92.2 to 119.24%, and the correlation coefficients (R^2^) were larger than 0.94 (Table 2). The Ct values of the twelve candidate reference genes ranged from 19.87 to 34.78 and covered all experimental conditions (Fig. 2). While most Ct values ranged from 19 to 27, *Actin, eEF2, β-Tubulin*, and *RPS3* were the most abundant transcripts under almost all experimental conditions. The least frequently expressed reference genes were *NADH, RPL17*, and *HSP83*. The five remaining reference genes were expressed at moderate levels.

### Stability of candidate reference genes

Four different algorithms (geNorm, NormFinder, BestKeeper, and delta-CT) were used to find stable reference genes. Gene expression stability was assessed under different experimental conditions such as biotic factors (Table 1; “Section Biotic factors (life stages and tissue types)”) and abiotic factors (“Section Abiotic factors (temperature, JHIII treatment and wild beetles vs lab-reared beetles)” using the web-based tool RefFinder.

#### Biotic conditions

**Figure 3:**
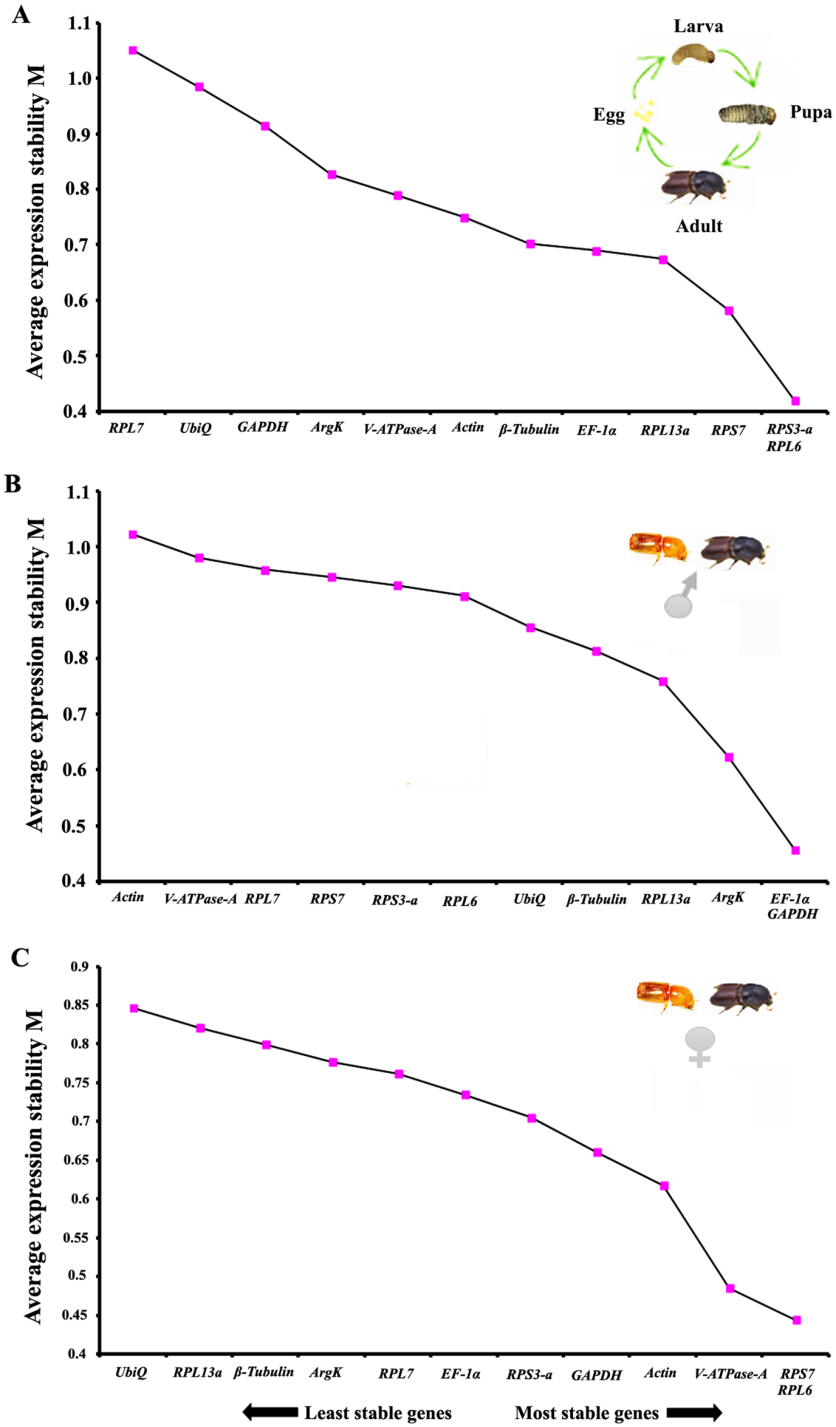
The average expression stability values (M) of twelve reference genes under different conditions (developmental stage and sex-specific conditions) were calculated by geNorm displaying the least stable (left) to the most stable (right). (**A**) Developmental stages (or life stages), (**B**) callow and fed adult male, (**C**) callow and fed adult female.

For different developmental stages that include three larval stages (from the first instar to third instar; L1–L3), one pupal stage (P), and two adults (callow and fed), the top three most stable candidates were *RPS3-a, EF-1a*, and *RPS7* based on Ct method, Bestkeeper, RefFinder and Normfinder (Table 3). The stability ranking order of the first three most stable reference genes that was obtained from four programs, was inconsistent (Table 3). The geNorm ranking of the top most stable reference genes was *RPS3-a*, *RPL6*, *RPS7*, and *RPL13a* (Fig. 3A). Integrating the results of all programs identified the consensus top three candidates, *RPS3-a, EF-1α*, and *RPS7,* as the most stable reference genes across the developmental stages. Alternatively, *GADPH, UbiQ*, and *RPL7* were the least stable genes (Table 3 and Fig. 3A).

**Table 3:** Ranking of the candidate reference genes based on stability values performed by Delta Ct, BestKeeper, RefFinder, and NormFinder, in life stages (or developmental stages) and sex-specific conditions.

Conditions	Genes	ΔCt Method		Best keeper		RefFinder		NormFinder		Recommended Genes
		Stability	Rank	Stability	Rank	Stability	Rank	Stability	Rank	
**Life stage**	*RPS3-a*	1.25	1	1.55	5	1.5	1	0.238	2	*RPS3-a, EF-1α* and *RPS7*
	*EF-1α*	1.31	2	1.64	7	3.44	3	0.245	3	
	*RPS7*	1.32	3	1.48	4	3.22	2	0.274	6	
	*RPL13a*	1.32	4	1.63	6	4.43	7	0.894	12	
	*RPL6*	1.35	5	1.75	9	4.05	4	0.224	1	
	*V-ATPase-A*	1.38	6	1.25	2	4.36	6	0.309	7	
	*ArgK*	1.58	7	0.93	1	4.3	5	0.264	5	
	*RPL7*	1.77	8	1.74	8	8	9	0.382	10	
	*β-Tubulin*	1.88	9	2.14	11	9.46	10	0.316	8	
	*Actin*	2.02	10	1.4	3	7.4	8	0.258	4	
	*UbiQ*	2.04	11	2.28	12	11.24	11	0.326	9	
	*GAPDH*	2.07	12	1.85	10	11.47	12	0.403	11	
**Male**	*RPS3-a*	1.2	1	1.12	6	1.57	1	0.246	1	*RPS3-a, RPL7* and *EF-1α*
	*RPL7*	1.29	2	0.91	2	2.38	2	0.295	8	
	*EF-1α*	1.38	3	1.22	8	4.56	4	0.316	11	
	*RPL6*	1.4	4	1.5	10	5.62	6	0.248	2	
	*GAPDH*	1.42	5	1.47	9	5.96	8	0.271	5	
	*RPL13a*	1.43	6	1.1	4	3.46	3	0.323	12	
	*RPS7*	1.47	7	1.21	7	5.86	7	0.250	3	
	*β-Tubulin*	1.51	8	0.83	1	4.6	5	0.308	9	
	*UbiQ*	1.72	9	1.07	3	6.84	9	0.314	10	
	*ArgK*	1.77	10	1.53	11	10.24	11	0.294	7	
	*Actin*	2	11	2.09	12	11.24	12	0.258	4	
	*V-ATPase-A*	2.08	12	1.11	5	9.64	10	0.285	6	
**Female**	*RPS3-a*	1.16	1	1.52	8	1.68	1	0.378	9	*β-Tubulin, RPS3-a* and *RPL6*
	*RPL7*	1.34	6	1.58	9	5.8	6	0.378	10	
	*EF-1α*	1.29	5	1.4	6	5.69	5	0.314	4	
	RPL6	1.18	2	1.71	10	2.78	2	0.318	5	
	*GAPDH*	1.34	7	1.73	11	7.8	10	0.248	1	
	*RPL13a*	1.46	10	1.11	3	7.21	9	0.342	7	
	RPS7	1.19	3	1.41	7	3.6	4	0.382	11	
	*β-Tubulin*	1.23	4	0.89	2	3.56	3	0.330	6	
	*UbiQ*	1.35	8	1.33	4	6.26	8	0.289	3	
	*ArgK*	1.45	9	1.38	5	7.98	11	0.263	2	
	*Actin*	1.87	12	2.37	12	12	12	0.364	8	
	*V-ATPase-A*	1.7	11	0.76	1	6.04	7	0.393	12

For sex-specific tissue comparisons (head, fat body, gut tissues of male and female beetles), the consensus top three candidates were calculated separately for male and female [callow male (CM), female (CF), fed adult male (AMF) and female (AFF)]. The top three most stable genes in male tissues were *RPS3-a, EF-1a*, and *RPS7*. The least stable genes defined by four programs were *V-ATPase-A, Actin*, and *ArgK* (Table 3). However, based on RefFinder, the most stable reference genes were *EF-1α, GADPH, ArgK*, and *RPL13a,* and the least were *Actin, V-ATPase-A,* and *RPL7* (Fig. 3B). In contrast, *β-Tubulin, RPS3-a,* and *RPL6* were the most stable genes in females. The least stable genes were *V-ATPase-A, Actin*, and *RPL7* (Table 3). The stability ranking for most stable reference genes was constant for females, i.e., *RPS7* and *RPL6* in the RefFinder (Fig. 3C).

**Figure 4:**
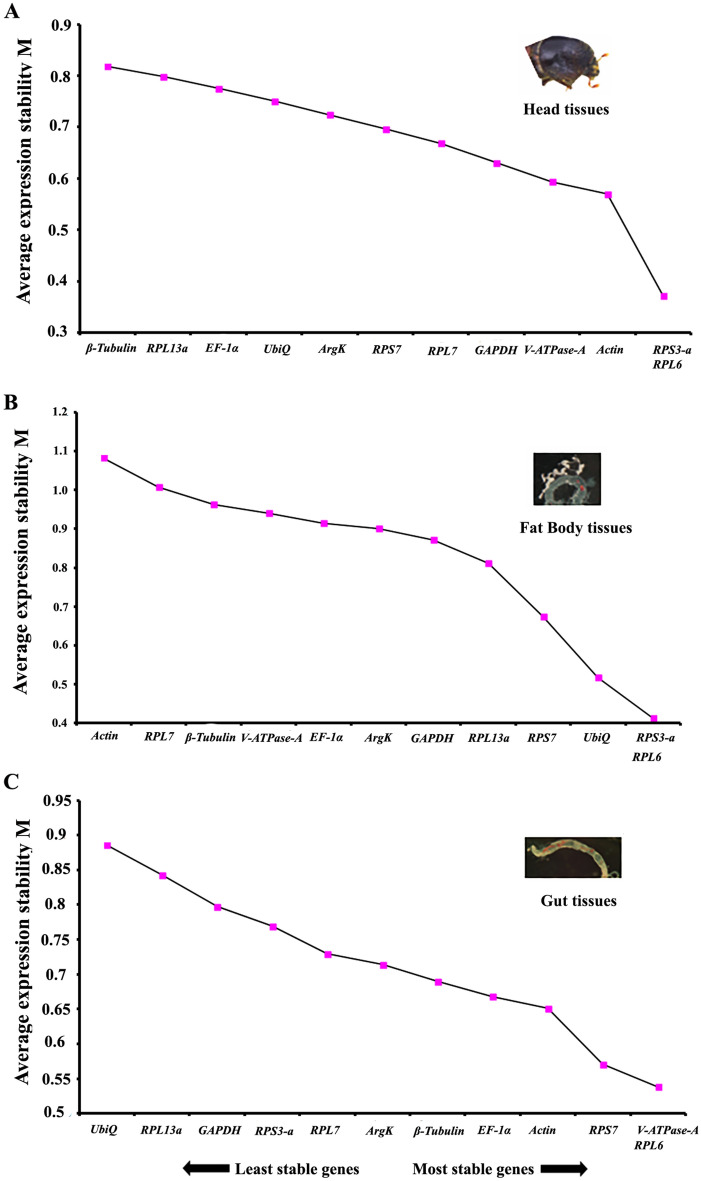
The average expression stability values (M) of twelve reference genes under different tissues calculated by geNorm demonstrating the least stable (left) to the most stable (right). (**A**) head, (**B**) fat body, (**C**) gut.

Tissue-specific expression stability of candidate reference genes was calculated for various bark beetle tissues (head, gut, and fat body). In head tissues, the most stable genes were *RPL7, RPL6*, and *RPS3-a* (Table 4). *RPS3-a* and *RPL6* were the best combinations of reference genes based on RefFinder (Fig. 4A). Similarly, *RPS7*, *GADPH*, *ArgK* and *EF-1α*, *RPS3-a,* *RPL6* were the most stable genes within the fat body and gut tissues, respectively (Table 4). In contrast, the best combination of the genes was *RPS3-a*/*RPL6* and *V-ATPase-A*/*RPL6,* respectively, as per RefFinder (Fig. 4B,C)*.* The least stable genes defined by four programs were *RPL13a, RPL7*, and *UbiQ* in the head, fat body, and gut tissues (Table 4). One interesting unanimous observation was that *RPS3-a *was the most stable reference gene except for the fat body under biotic conditions.

**Table 4:** Ranking of the candidate reference genes based on stability values performed by Delta Ct, BestKeeper, RefFinder, and NormFinder in tissue-specific stages.

Conditions	Genes	ΔCt Method		Best keeper		RefFinder		NormFinder		Recommended Genes
		Stability	Rank	Stability	Rank	Stability	Rank	Stability	Rank	
**Head**	*RPL7*	1.02	1	0.63	2	1.68	1	0.242	3	*RPL7*, *RPL6* and *RPs3-a*
	*RPL6*	1.03	2	0.74	3	1.86	2	0.300	11	
	*Actin*	1.09	3	1	9	3.95	5	0.258	4	
	*RPS3-a*	1.12	4	0.79	4	2.83	3	0.288	9	
	*β-Tubulin*	1.16	5	0.5	1	3.87	4	0.284	8	
	*UbiQ*	1.18	6	0.89	8	6.93	6	0.279	7	
	*ArgK*	1.23	7	1.07	10	7.65	7	0.261	5	
	*EF-1α*	1.27	8	1.16	11	7.7	8	0.314	12	
	*GAPDH*	1.31	9	1.43	12	8.97	10	0.186	1	
	*V-ATPase-A*	1.35	10	0.83	6	8.57	9	0.228	2	
	*RPS7*	1.44	11	0.8	5	9.03	11	0.270	6	
	*RPL13a*	1.71	12	0.83	7	10.49	12	0.293	10	
**Fat body**	*RPL7*	1.49	12	1.31	8	10.84	12	0.464	11	*RPS7, GAPDH* and *ArgK*
	*RPL6*	1.13	7	1.43	12	8.76	9	0.356	5	
	*Actin*	1.47	11	1.29	7	9.82	11	0.478	12	
	*RPS3-a*	1.07	4	1.29	6	5.42	8	0.387	8	
	*β-Tubulin*	1.18	9	0.86	1	4.74	6	0.405	9	
	*UbiQ*	1.18	10	1.37	10	9.46	10	0.296	2	
	*ArgK*	1.08	5	0.99	3	3.08	3	0.336	4	
	*EF-1α*	1.07	3	1.39	11	4.46	5	0.368	7	
	*GAPDH*	1.03	1	1.32	9	2.71	2	0.252	1	
	*V-ATPase-A*	1.16	8	1.07	4	4.12	4	0.428	10	
	*RPS7*	1.04	2	0.9	2	2.21	1	0.364	6	
	*RPL13a*	1.11	6	1.09	5	5.23	7	0.314	3	
**Gut**	*RPL7*	1.02	4	1.19	8	5.26	5	0.314	6	*EF-1α, RPS3-a* and *RPL6*
	*RPL6*	1.05	5	1.16	7	5.14	4	0.283	4	
	*Actin*	1.32	12	1.69	12	12	12	0.248	2	
	*RPS3-a*	0.9	1	1.06	4	1.41	1	0.329	7	
	*β-Tubulin*	1.09	7	0.94	3	5.66	7	0.249	3	
	*UbiQ*	1.21	9	1.36	11	9.46	11	0.364	10	
	*ArgK*	1.31	11	0.68	2	7.18	9	0.297	5	
	*EF-1α*	0.95	2	1.08	5	2.78	2	0.228	1	
	*GAPDH*	0.99	3	1.24	10	3.08	3	0.336	8	
	*V-ATPase-A*	1.29	10	0.46	1	5.62	6	0.352	9	
	*RPS7*	1.06	6	1.23	9	6.34	8	0.372	11	
	*RPL13a*	1.1	8	1.09	6	7.44	10	0.412	12

#### Abiotic conditions

**Figure 5:**
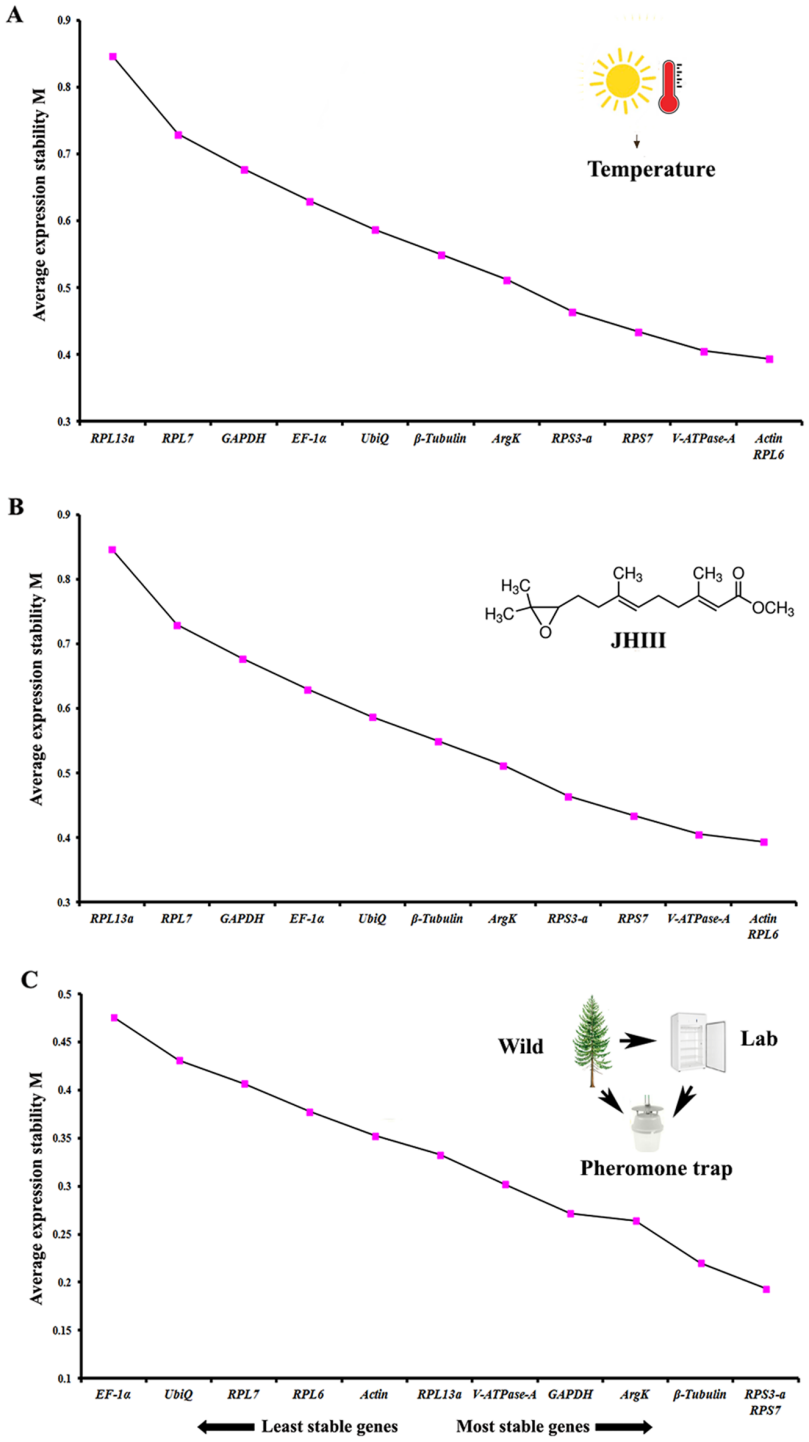
The average expression stability values (M) of twelve reference genes under different conditions calculated by geNorm where the least stable (left) to the most stable (right). (**A**) Temperature, (**B**) Juvenile hormone III, (**C**) Laboratory-reared vs wild beetles.

Abiotic conditions such as temperature, juvenile hormone III (JHIII) treatment, and laboratory-reared vs wild beetles were considered to evaluate the suitable reference genes using the aforementioned algorithms. After different temperatures incubations (4, 27, and 37 °C), *RPS3-a, RPS7*, and *V-ATPase-A* were documented as the most stable reference gene from four algorithms (Table 5). The best combination genes with the lowest expression stability value (M) or highest expression stability for different temperature exposure were *RPS3-a and RPS7* (Fig. 5A). The top three stable genes for juvenile hormone III treatment were *EF-1a, RPS7*, and *UbiQ* (Table 5). Based on RefFinder comprehensive ranking, *Actin, RPL6* and *V-ATPase-A* (Fig. 5B) were the most stable reference genes. The most stable reference gene expressions between wild and laboratory-reared beetles were *EF-1a, RPS3-a,* and *RPL13a* (Table 5), whereas the top three most stable reference genes via RefFinder were *RPL13a, RPL7 and RPS3-a* (Fig. 5C).

**Table 5:** Under the influence of abiotic factors, the candidate reference genes were ranked based on stability values performed by Delta Ct, BestKeeper, RefFinder, and NormFinder. The abiotic factors were temperature (temp), JHIII treatment, lab-reared vs wild-collected beetles.

Conditions	Genes	ΔCt Method		Best keeper		RefFinder		NormFinder		Recommended Genes
		Stability	Rank	Stability	Rank	Stability	Rank	Stability	Rank	
**Temp**	*RPS3-a*	0.56	1	0.97	6	1.57	1	0.15	7	*V-ATPase-A*, RPS3-a and *RPS7*
	*RPS7*	0.6	2	1.01	7	2.74	2	0.07	11	
	*UbiQ*	0.61	3	1.07	8	3.83	4	0.24	9	
	*V-ATPase-A*	0.61	4	0.82	2	2.99	3	0.15	10	
	*EF-1α*	0.63	5	1.25	11	5.76	6	0.28	1	
	*GAPDH*	0.67	6	1.12	9	6.64	7	0.08	5	
	*ArgK*	0.72	7	0.57	1	4.3	5	0.14	3	
	*β-Tubulin*	0.73	8	1.2	10	9.16	11	0.12	8	
	*RPL13a*	0.76	9	0.97	5	8.19	10	0.19	2	
	*Actin*	0.77	10	0.82	3	7.02	8	0.19	6	
	*RPL7*	0.78	11	0.89	4	7.89	9	0.20	4	
	*RPL6*	0.8	12	1.37	12	12	12	0.21	12	
**JHIII**	*EF-1α*	0.86	1	0.76	6	2.21	1	0.37	1	*EF-1α*, *RPS7* and *UbiQ*
	*RPS3-a*	0.87	2	0.84	7	3.03	4	0.23	7	
	*UbiQ*	0.9	3	0.73	4	3.66	5	0.27	9	
	*RPS7*	0.91	4	0.69	2	2.38	2	0.14	11	
	*RPL6*	0.92	5	0.71	3	2.94	3	0.18	12	
	*V-ATPase-A*	0.93	6	0.97	9	6.64	8	0.22	10	
	*GAPDH*	0.99	7	0.73	5	6.44	7	0.33	5	
	*β-Tubulin*	1.08	8	0.97	10	8.71	9	0.34	8	
	*ArgK*	1.09	9	0.63	1	5.05	6	0.32	3	
	*RPL7*	1.22	10	1.02	11	10.24	10	0.37	4	
	*RPL13a*	1.74	11	1.23	12	11.24	12	0.48	2	
	*Actin*	1.78	12	0.96	8	10.84	11	0.26	6	
**Lab vs. Wild**	*RPS3-a*	0.77	1	0.89	7	2.14	2	0.12	7	*EF-1α, RPS3-a* and *RPL13a*
	*EF-1α*	0.79	2	0.81	4	2	1	0.22	1	
	*RPL13a*	0.81	3	0.98	9	4.82	5	0.19	2	
	*UbiQ*	0.83	4	0.96	8	4.43	4	0.24	9	
	*V-ATPase-A*	0.86	5	0.73	2	2.99	3	0.13	10	
	*RPL7*	0.9	6	1.06	10	6.77	8	0.16	4	
	*RPS7*	0.98	7	1.18	11	8.1	9	0.26	11	
	*RPL6*	1.02	8	1.29	12	8.49	10	0.22	12	
	*GAPDH*	1.05	9	0.74	3	6.18	7	0.23	5	
	*β-Tubulin*	1.33	10	0.68	1	5.62	6	0.17	8	
	*Actin*	1.41	11	0.83	5	9.03	11	0.23	6	
	*ArgK*	1.55	12	0.85	6	10.09	12	0.27	3

### Selection of optimal reference genes for normalization

**Figure 6:**
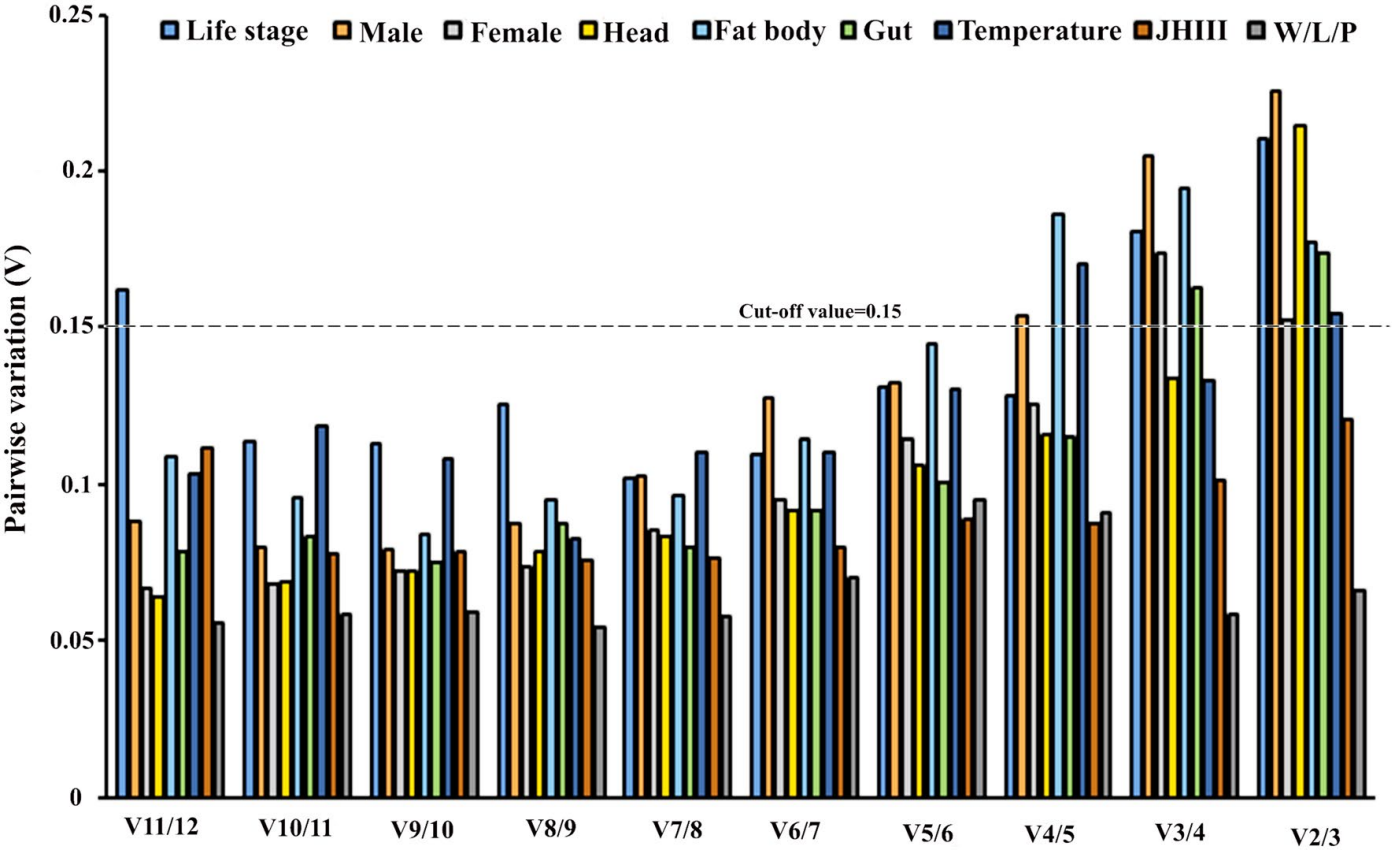
The optimal number of reference genes for the normalization of *I. typographus* under different experimental conditions [developmental stages, sex-specific (male and female), tissue-specific (head, midgut, fat body, temperature; Juvenile hormone III; wild-collected vs long-term laboratory-reared beetles]. Based on geNorm analysis, average pairwise variations were calculated between the normalization factors NFn and NFn + 1. Values < 0.15 imply that n + 1 genes were unnecessary to normalise gene expression.

To determine the consistent and more accurate results of the optimal number of reference genes in each experimental condition, pairwise variation (V) was assessed using geNorm^25^. The optimal number of reference genes by calculating pairing variable value Vn/n + 1 with a cut-off value of 0.15, where the n + 1 reference gene is unnecessary with values under the threshold. Alternatively, the first references gene is sufficient to normalize the target gene expression in those cases (Fig. 6). For biotic conditions minimum of two genes is required for normalization as per V-value above 0.15 in V2/3.

### Validation of reference gene selection

**Figure 7:**
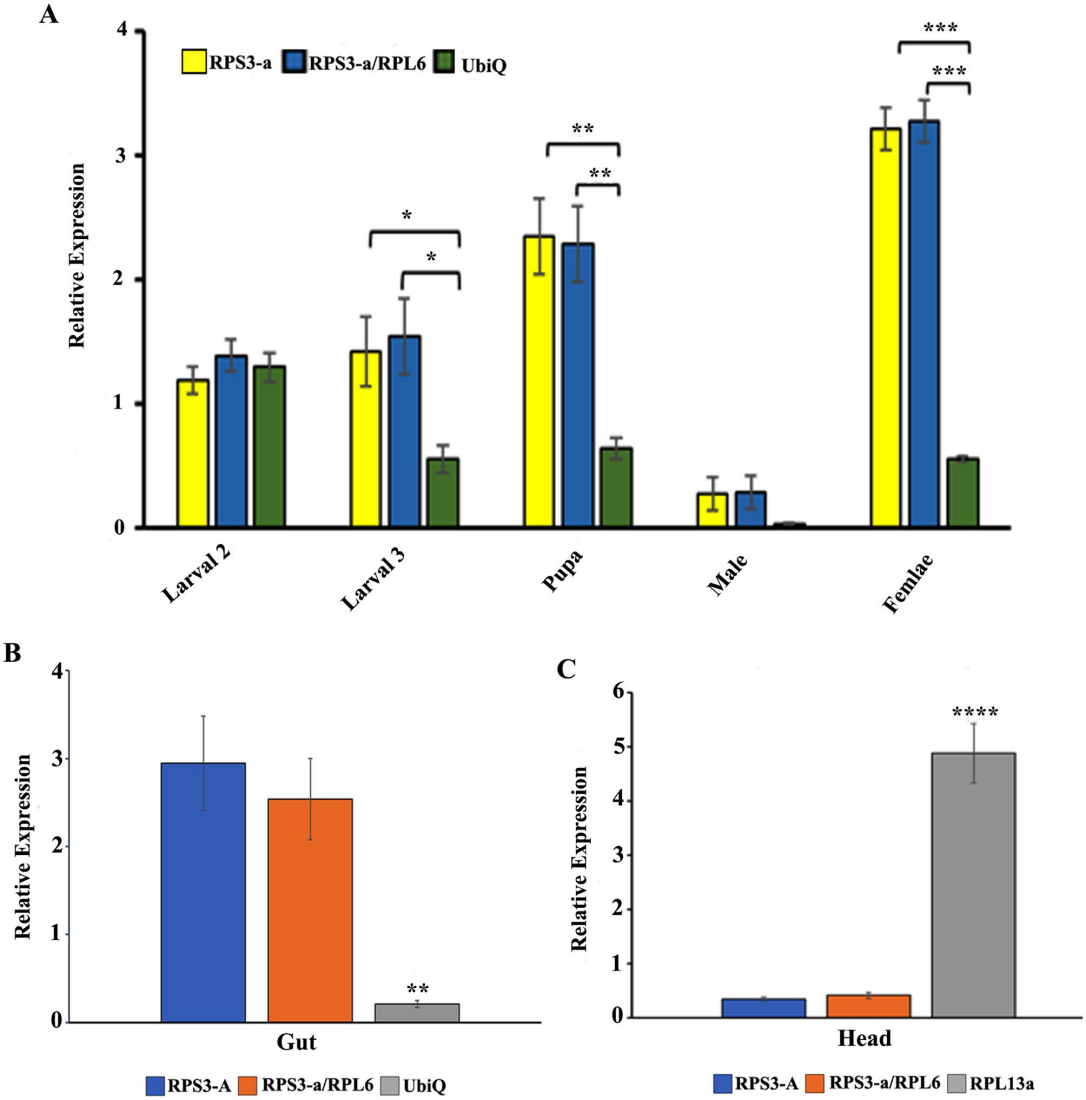
The relative expression levels of the target gene *CYP450* were studied under developmental and various tissues after normalizing with the least and most suitable reference genes separately or in combination. The most stable reference gene *RPS3-a* and *RPL6* for developmental stages, head, and gut tissue. The least stable reference gene *UbiQ* was used for both developmental and gut. For head tissue, *RPL13a* was used as the least stable reference gene. (**A**) Developmental stage, (**B**) Gut. (**C**) Head. Data represent mean values ± SD of four biological replicates. Asterisks indicate significant differences in the expression of the target gene normalized separately by different reference genes (****P < 0.0001***P < 0.001, **P < 0.01, *P < 0.05, ns indicate no significant difference).

To validate the best reference gene, the expression of *CYP450* in the developmental stages and tissue types were normalized with single reference genes and gene combinations recommended by geNorm (Fig. 6). The relative expression pattern of *CYP450* was normalized with the expression level of most stable (*RPS3-a, RPS3-a/RPL6*) and the least stable reference genes (*UbiQ* for gut, *RPL13a* for head tissues). The results showed that *CYP450* expression in third instar larva, pupa, and female tissues increased after normalization with the most stable reference gene alone or in combination (Fig. 7A). In comparison, when the least stable *UbiQ* was used as a reference gene, the *CYP450* expression patterns were inconsistent across different stages (Fig. 7A). Similarly, the expression patterns of *CYP450* after normalization based on the single (*RPS3-a*) and combined (*RPS3-a/RPL6*) genes expression in gut and head tissues were showed a similar pattern (Fig. 7B,C). On the contrary, *UbiQ* expression-based normalization reduced the expression of *CYP450* from 2.9 to 0.2-fold in the gut, whereas *RPL13a* based normalization increased 4.5-fold in head tissue. Hence, our result demonstrates the importance of selecting and validating reference genes to avoid misinterpretation of gene expression results.

## Discussion

RT-qPCR is one of the effective and reliable techniques for quantifying the expression of mRNA levels under different experimental conditions. However, multiple factors, such as RNA extraction, cDNA synthesis, primers, and materials handling, impact the RT-qPCR results. The reliable reference gene can overcome confounding variations in the data. Therefore, the stability of the reference gene must be evaluated for each experimental condition to accurate and reliable data interpretation^37,42,49,52,53^. Ideally, the reference gene should have a steady and unaltered expression level during the entire experimental conditions^25^. Hence, screening the stable reference genes in different experimental conditions before examining the target gene expression is necessary for working on any insect system.

Although *I. typographus* is one of the most destructive pests of Norway spruce (*Picea abies*)^1,3,4,54^, environmental stress such as drought significantly impacts its host colonization. It can provoke transitions from endemic to an epidemic bark beetle population, suitable for the mass attack to healthy trees^55,56^. Unravelling the mechanisms underlying *I. typographus* and host interaction was a daunting task until a decade ago. However, recent advances in genomic and transcriptomic studies on *I. typographus*^44^ have managed to demonstrate the power of gathering valuable genetic information in delineating the beetle-host interaction dynamics. However, it is just the beginning, and *w*ithout reference genes, there is no way forward for gene expression and RNAi based functional genomics studies. Our study delivers a catalogue of reference genes for the impending genomic and functional studies on *I. typographus*.

**Figure 8:**
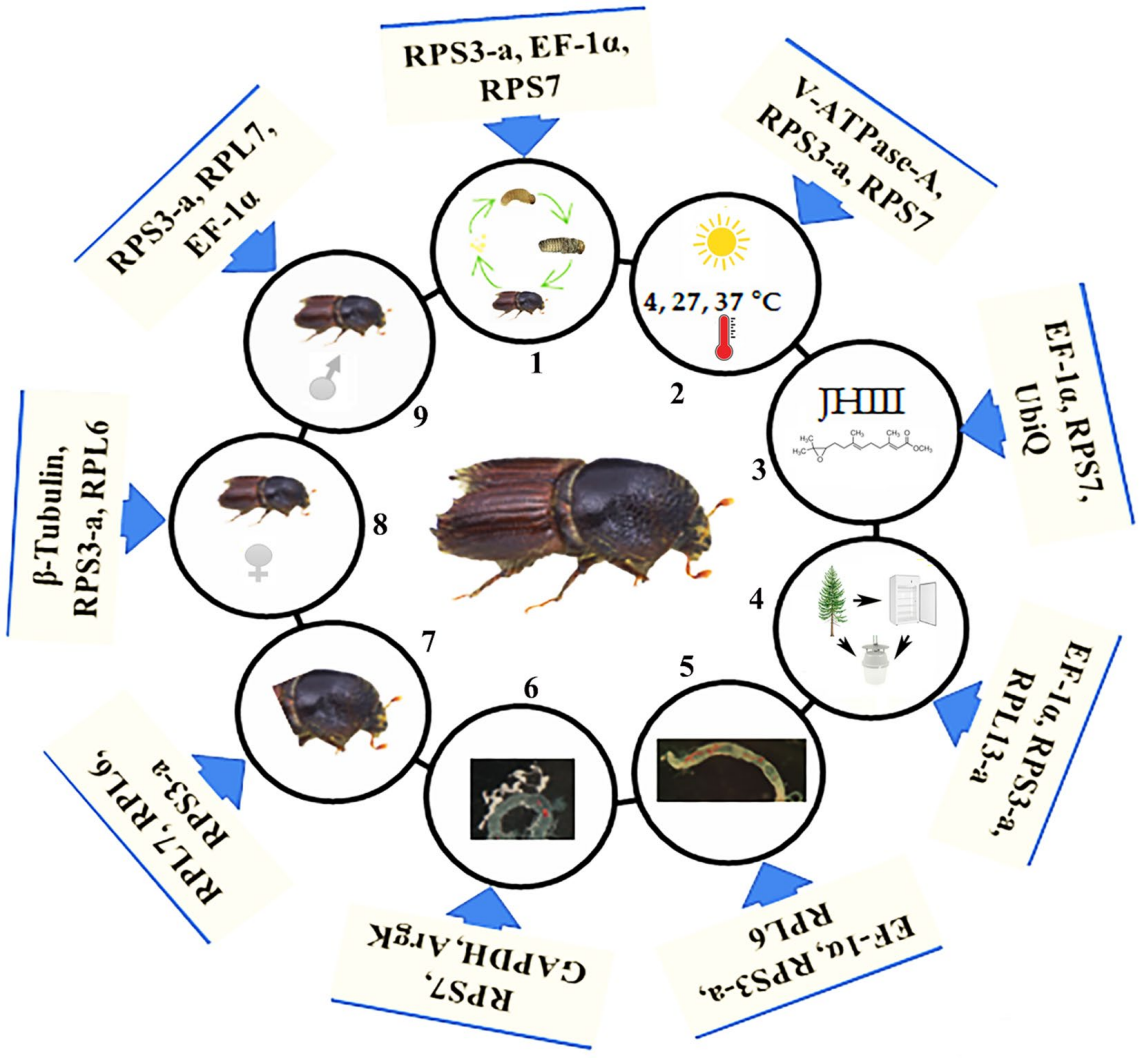
Recommended reference genes under different experimental conditions for *I. typographus*. 1. Developmental stages (or life stages). 2. Temperature treatment. 3. JHIII treatment. 4. Wild /lab-reared beetles/ pheromone traps. 5. Gut tissues. 6. Fat body tissues 7. Head tissues. 8. Female tissues. 9. Male tissues.

In this study, twelve candidates reference genes were tested for the first time in the bark beetle *I. typographus* across various experimental conditions, including developmental stage, sex-specific, tissues specific, and exposure to abiotic conditions (Tables 3, 4, 5; Figs. 3, 4, 5). The results showed that all reference gene primers acquire good amplification efficiency (92.2% to 119.24%) and regression coefficient (0.944 to 0.998). Among the twelve reference genes in this study, we found that *RPS3-a, EF-1α, RPL6, RPS7*, and *RPL7* were the most stable reference genes for *I. typographus* under various experimental conditions (Fig. 8).

*Ribosomal proteins* (RPs: *RPL*- Ribosomal protein large subunit and *RPS*- Ribosomal protein small subunit) are a large group of proteins that play a crucial role in the cellular process such as protein synthesis, cell growth, and development^57–59^ Lee et al.^60^ demonstrated that ribosomal mutation controls the cellular processes but not direct consequences of ribosome depletion. Several reports indicated that ribosomal protein was widely used as a reference gene in insect functional genomics^31,38,61^. Not surprisingly, ribosomal protein (*RPL* and *RPS*) was consistently expressed throughout most of the experimental conditions of the insect species. For example, *RPL10* exhibited the most stable expression in different tissues, different diets conditions, and populations of *Spodoptera litura*, whereas *RPS3* was expressed most stably in larvae after starving^62^. *RPS13* and *RPS7* exhibited the most stable expression under larval-crowding conditions in *Mythimna separata*^63^. Similarly, different developmental stages (*RPL32, RPS18*) and different tissues (*RPS18*) of *Spodoptera frugiperda* showed the most stable expression^64^, whereas *RPS26* and *RPL32* genes showed the same in *Thermobia domestica*^65^; *RPS11*, *RPL28*, and *RPL10* genes showed *Tuta absoluta*^58^; *RPS18* and *RPL13* genes showed *Rhopalosiphum padi* tissues^66^. Sellamuthu et al.^43^ reported that *RPS3* is stably expressed in the developmental stage, sex-specific and tissue-specific conditions in pine-feeding bark beetle, *I. sexdentatus*. Furthermore, *RPL36* displayed stable expression in the developmental stage, sex-specific and tissue-specific conditions of *Glenea cantor*^67^. Developmental stage and tissue-specific expression of *RPS18* and *RPS11* were highly stable in *Cicindela campestris*^36^. Similarly, *RP49* expression was the most stable in tissue and sex-specific conditions in *Harmonia axyridis*^33^*.* Wang et al. ^68^ reported that *RPL22e* was stably expressed in sex-specific conditions of *Mylabris cichorii. RPS23* and *RPL12* were identified as reference genes in *Anthonomus eugenii*^22^. Similarly, *RPL32* gene was found as the most stable gene in adult (male and female) tissues of *Anoplophora glabripennis*^69^*.* It was reported that *RPL13A* ribosomal protein was more stable expression under low-temperature treatments in *Thitarodes armoricanus*^70^ and *Paederus fuscipes*^71^. Other ribosomal proteins such as *RPL27* and *RPS7, RPL7* of *S. frugiperda*, were the most stable reference genes under low-temperature and high-temperature^64^. Tao et al.^72^ showed that *RPL-33* and *RPS-26* are the most stable reference genes from microarray data and *RPS-2* and *RPS-4* from RNA-seq of *Caenorhabditis elegans* out of thirteen ribosomal proteins. Moreover, *RPS20* was detected as the least stably expressed gene for analyzing *Plutella xylostella* under different conditions^73^. Our results also demonstrated stable ribosomal gene expression in biotic and abiotic conditions. According to developmental, sex-specific, and tissue-specific stages, *RPS3-a*, *RPL7*, and *RPS6* showed higher expression stability. Furthermore, *RPS3-a* and *RPS7* were stably expressed as second top rank in various abiotic conditions. These results also suggest that no single reference gene for different experimental conditions is observed in the present study.

*Elongation factor 1* (*EF-1*) plays a central role that promotes the delivery of aminoacyl-tRNA to the acceptor site of the ribosome during protein biosynthesis^32,74^. *EF-1* has been a stable reference gene for many years and is widely used for reference genes in many insects^61–63,70^. Our results exhibited that *elongation factor 1* was the second most stable expression in biotic and abiotic conditions. Similarly, Su et al.^67^ reported that *EF-1* was the most appropriate reference gene for all samples of *Glenea cantor*. Teng et al. ^75^ reported *elongation factor2* (*eEF2*) as the most stably expressed gene in different developmental stages of *Plutella xylostella*. The expression stability of *eEF2* in developmental stages and tissues was also documented in *Agrilus planipennis*^76^; *I. sexdentatus*^43^; *Sogatella furcifera*^77^; *Mythimna separata*^63,78^; *Tuta absoluta*^61^; *Hippodamia variegate*^42^; *Spodoptera frugiperda*^64^. However, Ponton et al. ^32^ reported that the elongation factor was unsuitable for normalization of relative expression due to the different expression variability in different experimental conditions in *Drosophila melanogaster*. Nevertheless, our results confirmed that *EF-1α* had sable expression between JHIII treated vs control bark beetles, wild vs lab-reared beetles, and within gut tissues, male tissues, and developmental stages.

To further validate our findings and confirm the stable reference gene, we analyzed the expression of cytochrome P450 (*CYP03903*) in the developmental stages, head, and gut tissues of the adult beetle. P450s is a large class of enzymes that often plays an essential role in detoxifying xenobiotics^79^. It evolves during the insect-host interaction to metabolize a wide range of plant allelochemicals^80^. Our results demonstrated the expression trends in developmental and tissue-specific conditions using single reference gene or gene combinations. The *CYP03903* transcript was increased in pupa and female tissue compared to other stages. However, the same expression profile of *CYP03903* significantly differs when normalized with the least stable reference gene showing the importance of having a suitable reference gene. For instance, the expression of CYP*450* demonstrated just the opposite pattern based on the most or least stable gene was used for normalization. Nevertheless, the *CYP03903* expression profile between the gut and head tissues is different, suggesting different functions in the respective tissues^79,81^.

To summarise the present study, twelve candidate reference genes of *I. typographus* were selected and systematically evaluated for their expression stability using four widely used software programs under different experimental conditions to obtain the best reference genes for each condition. The results showed that the most suitable candidate reference genes were *RPS3-a, EF-1a*, *RPS7, RPL7,* and *RPL6* under different experimental conditions. Based on our comprehensive analysis, we recommended a list of reference genes and combinations of reference genes be used to normalize gene expression in *I. typographus* subjected to different experimental conditions. This is a much-awaited reference gene validation work on *I. typographus,* setting the foundation for future molecular (i.e., gene expression) and functional genomics studies (i.e., RNAi). The same genes can be further evaluated to identify suitable reference genes for other *Ips* species (Coleoptera: Curculionidae: Scolytinae).

## Supplementary Information

Supplementary Information 1.

Supplementary Information 2.

## Data Availability

The raw data supporting the conclusions of this manuscript will be made available by the authors.
