# Supplementary figures and images for: Identifying optimal reference genes for gene expression studies in Eurasian spruce bark beetle, Ips typographus (Coleoptera: Curculionidae: Scolytinae)
Source: Sci Rep. 2022 Mar 18;12:4671. doi: 10.1038/s41598-022-08434-3 (PMC8933438; doi:10.1038/s41598-022-08434-3)

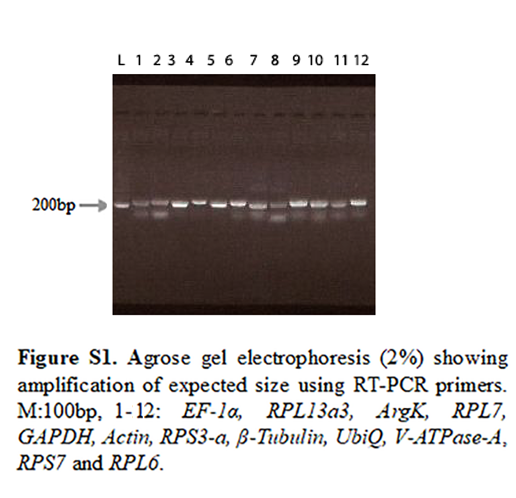

Supplement: Supplementary file 1 — Supplementary Information 1. [file 41598_2022_8434_MOESM1_ESM.tif]

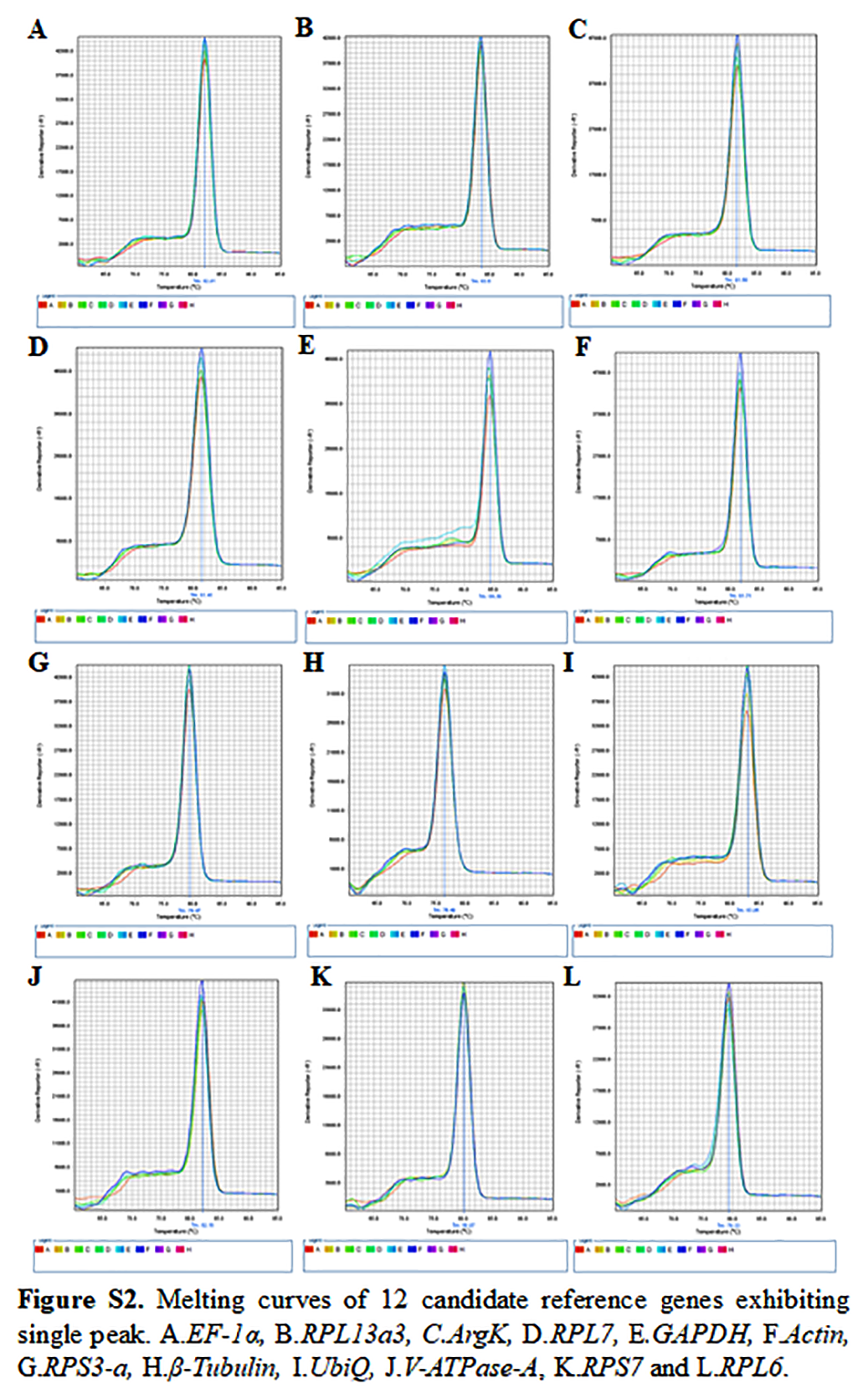

Supplement: Supplementary file 2 — Supplementary Information 2. [file 41598_2022_8434_MOESM2_ESM.tif]
